# How does lymph node yield affect survival outcomes of stage I and II colon cancer?

**DOI:** 10.1186/s12957-020-1802-6

**Published:** 2020-01-29

**Authors:** Chi Chung Foo, Clement Ku, Rockson Wei, Jeremy Yip, Julian Tsang, Toi Yin Chan, Oswens Lo, Wai Lun Law

**Affiliations:** 1 Department of Surgery, University of Hong Kong, Hong Kong, China; 2 Department of Surgery, Queen Mary Hospital, Hong Kong, China

**Keywords:** Lymph node, Colon cancer, Survival

## Abstract

**Background:**

According to the American Joint Committee on Cancer staging for cancer of the colon, a minimum of 12 lymph nodes (LN) has to be sampled for accurate staging. This has bearing on the long-term prognosis and the need for adjuvant chemotherapy. The aim of this study was to revisit the association of lymph node yield and the long-term survival in patients with stages I and II, i.e. node-negative, colon cancer.

**Method:**

Consecutive patients who underwent elective or emergency curative resections for cancer of colon between the years 2003 and 2012 were retrospectively reviewed. Only patients with stage I or II diseases (AJCC 8th edition) were included. They were analysed in three groups, LN<12, LN12-19 and LN≥20. Their clinic-pathological characteristics were compared. The disease-free (DFS) and overall survival (OS) were estimated with the Kaplan-Meier method and compared with the log-rank test.

**Results:**

There was a total of 659 patients included in the analysis. Twelve or more LN were found in 65.6% of the specimens. The mean follow-up was 83.9 months. LN≥20 had significantly better DFS (*p* = 0.015) and OS (*p* = 0.036), whereas LN<12 had similar DFS and OS when compared to LN12-19. The advantage in DFS and OS were mainly seen in those with stage II diseases. A lymph node yield of greater than 20 was one of the predictors of favourable DFS, hazard ratio 0.358; 95% CI 0.170–.756, *p* = 0.007.

**Conclusion:**

The lymph node yield had a significant association with survival outcomes. A lymph node yield of 20 or more was associated with better survival outcomes. On the other hand, lymph node yield less than 12 was not shown to have inferior survival outcomes when compared to those between 12 and 19.

## Background

Staging is an integral part of colon cancer treatment. The widely adopted staging system, TNM staging maintained by the American Joint Committee on Cancer (AJCC) and the International Union for Cancer Control (UICC), requires at least 12 lymph nodes to be harvested for adequate staging [1]. The minimum number of 12 was proposed in the early 1990s and was based on a study that suggested this was enough to determine node positivity in 94% of the specimens [2]. However, the discussion went on as to what should be the optimal lymph node yield [3]. Studies also suggested that lymph node yield might serve as a prognostic indicator [4, 5]. The technique of complete mesocolic excision, which involves the removal of the mesocolon in its intact envelope and the ligation of the central vascular pedicle, yet again draws the attention of lymph node clearance in colon cancer surgery [6]. This study aimed to revisit the association of lymph node yield and the long-term survival in patients with stages I and II, i.e. node-negative, colon cancer. The hypothesis of this study was that inadequate lymph node yield would have an adverse effect on survival outcomes and that lymph node yield more than the suggested 12 lymph nodes would have similar survival outcomes.

## Method

This was a retrospective study of consecutive patients who underwent colonic resection of curative intent for adenocarcinoma of colon in an academic hospital within the study period from the year 2003 to 2012. Patient demographics, operative information and pathology data were kept in a prospectively maintained database. Those who had stage I or stage II disease were included in the analysis. This study was approved by the institutional review board.

During the study period, routine central vascular pedicle ligation was not practised in right colectomies. For left-sided resections involving the ligation of the inferior mesenteric artery, high ligation was routinely performed unless the surgeon deemed inappropriate. When there were less than 12 lymph nodes sampled, the pathologist would be asked to re-examine the specimen for more.

Patients were followed up every 3 months in the first 2 years, every 4 to 6 months from the third to the fifth year and then yearly thereafter. Clinical examination was performed and the carcino-embryonic antigen level was checked during follow-up visits. Contrast-enhanced computed tomography of the thorax, abdomen and pelvis was performed yearly during the first 3 years and when indicated thereafter. Patients who were operated for colon cancer were routinely followed until death. The survival status of patients was traced from the public hospital central electronic health system, as the death of patients within the territory would be registered in the system regardless of cause and place of death. Follow-up time and time to recurrence or death were counted from the date of operation.

Patients were analysed according to the number of lymph nodes harvested from the specimen. They were divided into three groups, < 12 (LN<12), 12 to 19 (LN12-19) and ≥ 20 (LN≥20). Twenty was chosen as the cut off as a previous study showed no significant survival benefit beyond this number [7]. The difference between these groups was analysed with the *χ*^2^ test for categorical variables and the one-way analysis of variance (ANOVA) test for continuous variables. Survival analysis was done with the Kaplan-Meier estimate method. The overall (OS) and disease-free survival (DFS) of these groups were compared with the log-rank test. Univariate analysis of various clinic-pathological parameters using the survival outcomes as the dependent factor was performed with the Cox-Mantel log-rank test. A hazard ratio of greater than 1 signifies poorer survival outcomes. Significant parameters, those with *p* value < 0.05, were included in the multivariate analysis using the Cox regression analysis in a backward stepwise method. All statistical calculations were performed with the SPSS version 23 (IBM, USA).

## Results

### Patient cohort

There was a total of 659 patients included in this study, with 23.7% and 76.3% having stage I and stage II disease, respectively. The number of lymph nodes harvested ranged from 0 to 86, with a mean of 15.2. There were 12 or more lymph nodes in 65.6% of the specimens. The mean follow-up was 83.9 months. There were 89 (13.5%) patients who suffered from disease recurrence. Table 1 summarized the demographic characteristics of this study cohort.

**Table 1:** The clinic-pathological characteristics of patients in various lymph node yield groups

	Total	LN<12	LN12-19	LN≥20	*p*
	*N* = 659	*N* = 227	*N* = 268	*N* = 164	
Age at surgery	71.8 ± 12.1	72.1 ± 11.46	73.42 ± 11.14	68.51 ± 13.81	< 0.001
Gender					
Male	363 (55.1%)	132 (58.1%)	155(57.89%)	76(46.3%)	0.034
Female	296 (44.9%)	95(41.9%)	113(42.2%)	88 (53.7%)	
ASA					
1–2	431 (65.4%)	144(66.1)	166(63.6)	121(80.1)	0.002
≥ 3	199 (30.2%)	74(33.9)	95(36.4%)	30(19.9%)	
CCI	2.47 ± 0.8	2.4 ± 0.8	2.6 ± 0.8	2.4 ± 0.7	0.105
Preoperative CEA	12.5 ± 40.6	8.7 ± 30.6	13.1 ± 39.1	16.7 ± 53.3	0.245
Year of surgery					
2003–2007	329 (49.9%)	151 (66.5%)	110 (41.0%)	68 (41.5%)	< 0.001
2008–2012	330 (50.1%)	76 (33.5%)	158 (59.0%)	96 (58.5%)	
Type of surgery					
Elective	555 (84.2%)	187 (82.4%)	231 (86.2%)	137 (83.5%)	0.491
Emergency	104 (15.8%)	40 (17.6%)	37 (13.8%)	27 (16.5%)	
Surgical approach					
Laparoscopic	304 (46.1%)	90 (39.8%)	137 (51.3%)	77 (47.0%)	0.011
Open	335 (50.8%)	134 (59.3%)	121(45.3%)	80 (48.8%)	
Laparoscopic converted open	18 (2.7%)	2 (0.9%)	9 (3.4%)	7 (4.3%)	
Site of tumour					
Caecum	72 (10.9%)	24 (10.6%)	31 (11.6%)	17 (10.4%)	0.914
Ascending colon	97 (14.7%)	28 (12.3%)	45 (16.8%)	24 (14.6%)	
Hepatic flexure	63 (9.6%)	17 (7.5%)	27 (10.1%)	19 (11.6%)	
Transverse colon	93 (14.1%)	33 (14.5%)	37 (13.8%)	23 (14.0%)	
Splenic flexure	31 (4.7%)	10 (4.4%)	11 (4.1%)	10 (6.1%)	
Descending colon	67 (10.2%)	28 (12.3%)	26 (9.7%)	13 (7.9%)	
Sigmoid colon	236 (35.8%)	87 (38.3%)	91 (33.9%)	7 (35.3%)	
Type of resection					
Right sided	323 (49.0%)	99 (43.6)	139 (51.9%)	85 (51.8%)	0.159
Left sided	306 (46.4%)	120 (52.9%)	117 (43.7%)	69 (42.1%)	
Subtotal	30 (4.6%)	8 (3.5%)	12 (4.5%)	10 (6.1%)	
Perforation					
Yes	29 (4.4%)	10(4.4%)	13(4.9%)	6(3.7%)	0.842
No	630 (95.6%)	217(95.6%)	255(95.1%)	158(96.3%)	
Obstruction					
Yes	114 (17.3%)	37(16.4%)	44(16.4%)	33 (20.2%)	0.531
No	543 (82.4%)	189(83.6%)	224(83.6%)	130(79.8%)	
Tumour size	42.1 ± 26.6	37.1 ± 21.9	42.6 ± 27.5	48.0 ± 29.7	< 0.001
T stage					
1&2	155 (23.6%)	68 (30.0%)	61 (22.8%)	26 (15.9%)	0.005
3&4	503 (76.4%)	159 (70.0%)	206 (77.2%)	138 (84.1%)	
Differentiation					
Well	58 (8.8%)	24 (11.3%)	24 (9.2%)	10 (6.2%)	0.563
Moderate	533 (80.9%)	176 (83.0%)	216 (83.1%)	141 (87.6%)	
Poor	39 (5.9%)	11 (5.2%)	19 (7.3%)	9 (5.6%)	
Perineural invasion					
Yes	40 (6.1%)	15 (6.8%)	15 (5.7%)	10 (6.1%)	0.888
No	604 (91.7%)	205 (93.2%)	246 (94.3%)	153 (93.9%)	
LVP					
Yes	92 (14.0%)	39 (17.3%)	39 (14.8%)	14 (8.6%)	0.049
No	559 (84.8%)	186 (82.7%)	225 (85.2%)	148 (91.4%)	
Mucinous					
Yes	63 (9.6%)	16(7.2%)	31 (11.7%)	16 (10.0%)	0.383
No	583 (88.5%)	205 (92.8%)	234 (88.0%)	144 (90.0%)	
Signet					
Yes	2 (0.3%)	221 (100%)	263 (99.2%)	160 (100.0%)	0.236
No	644 (97.7%)	0 (0%)	2 (0.8%)	0 (0%)	
Margin					
Involved	1 (0.4%)	0 (0.0%)	1 (0.6%)	2 (0.3%)	0.481
Clear	226 (99.6%)	268 (100.0%)	163 (99.4%)	657 (99.7%)	
Adjuvant chemotherapy					
Yes	36 (15.9%)	35 (13.1%)	35 (21.3%)	106 (16.1%)	0.075
No	191 (84.1%)	233 (86.9%)	129 (78.7%)	553 (83.9%)	

*ASA* American Society of Anaesthesiologist grade, *CCI* Charlson co-morbidity index, *CEA* carcino-embryonic antigen, *LVP* lymphovascular permeation

### Lymph node yield

The clinic-pathological parameters of the three lymph node yield groups were shown in Table 1. Female gender (*p* = 0.034), younger age (*p* < 0.001), ASA 1 and 2 (*p* = 0.002), laparoscopic surgery (*p* = 0.011), T3 and T4 tumours (*p* = 0.005), negative lymphovascular permeation (*p* = 0.049), greater tumour size (*p* < 0.001) and those that were operated from 2008 to 2012 (*p* < 0.001) were associated with a higher lymph node yield.

### Disease-free survival

**Fig. 1:**
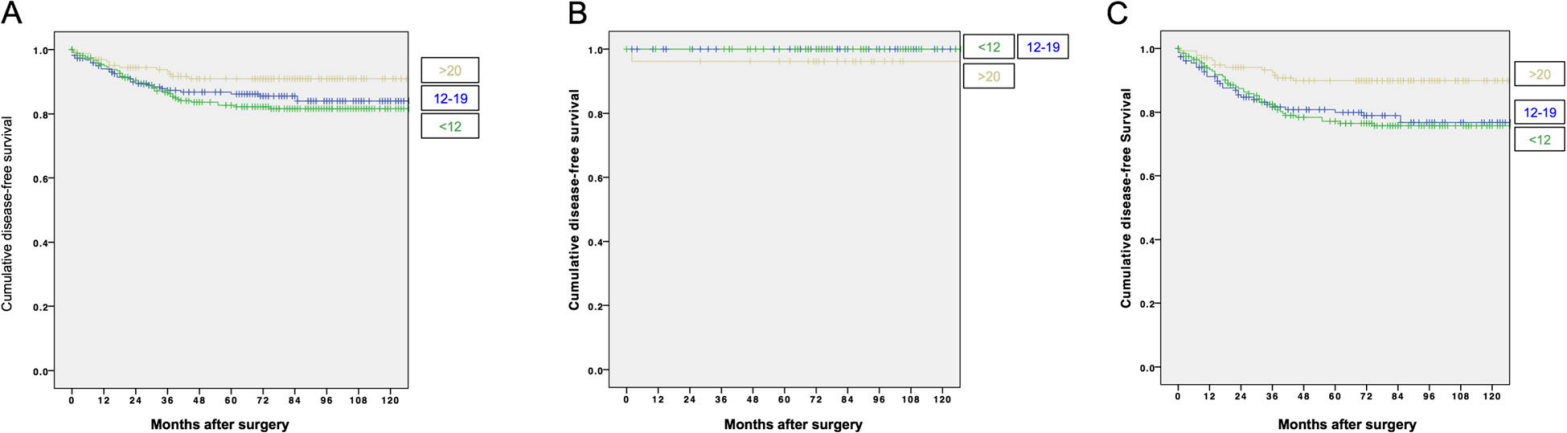
Disease-free survival curves for **a** overall, **b** stage I disease and **c** stage II disease

The DFS according to the lymph node yield was shown in Fig. 1 a. LN≥20 had significantly better DFS (*p* = 0.015) compared to the other two groups. Figure 1 b and c showed the DFS of stage I and II diseases, respectively. The survival benefit from a higher lymph node yield was mainly seen in patients with stage II diseases. The 5-year DFS was 86.2%, 82.7% and 91.0% for LN<12, LN12-19 and LN≥20, respectively (Table 2). The 10-year DFS was 84.0%, 81.6% and 91.0% for LN<12, LN12-19 and LN≥20, respectively.

**Table 2:** The survival outcomes of patients in various lymph node yield group

	Lymph node yield			*p*
	< 12	12–19	≥ 20	
Disease-free survival				
Stage I and II				0.015
5 years	86.2	82.7	91.0	
10 years	84.0	81.6	91.0	
Stage I				NA
5 years	100	100	96.2	
10 years	100	100	96.2	
Stage II				0.085
5 years	80.0	77.2	90.0	
10 years	76.8	75.8	90.0	
Overall survival				
Stage I and II				0.036
5 years	68.2	70.4	78.9	
10 years	44.8	51.2	57.5	
Stage I				0.878
5 years	83.8	81.4	88.5	
10 years	60.0	70.1	55.0	
Stage II				0.001
5 years	61.4	67.1	77.1	
10 years	38.5	46.2	57.6	

The association between various clinic-pathological parameters and the DFS were shown in Table 3. Among these, lymph node yield greater than 20 (HR 0.358; 95% CI 0.170–.756, *p* = 0.007), obstructed tumour (HR 2.061; 95% CI 1.128–3.767, *p* = 0.019), high preoperative CEA level (HR 1.004; 95% CI 1.000–1.008, *p* = 0.038), poorly differentiated tumour (HR 2.263; 95% CI 1.058–4.843, *p* = 0.035) and T stage ≥ 3 (HR 17.216; 95% CI 2.367–125.235, *p* = 0.005) were predictors of disease-free survival from the multivariate analysis.

**Table 3:** The univariate and multivariate analysis of various clinic-pathological factors, using disease-free survival as the dependent variable

	Univariate HR	*p*	*Multivariate HR	*p*
Age	1.009 (0.991–1.028)	0.314		
Male	1.309 (0.859–1.996)	0.211		
ASA ≥ 3	1.150 (0.729–1.815)	0.549		
Year of surgery 2003–2007	1.399 (0.919–2.130)	0.117		
Preoperative CEA	1.005 (1.002–1.009)	0.001	1.004 (1.000–1.008)	0.038
CCI	0.919 (0.689–1.224)	0.563		
Obstruction	2.403 (1.530–3.773)	< 0.001	2.061 (1.128–3.767)	0.019
Perforation	2.971 (1.436–6.145)	0.003		
Emergency surgery	2.549 (1.606–4.046)	< 0.001		
Laparoscopic approach	0.577 (0.377–0.884)	0.012		
Right-sided resection	1.117 (0.739–1.688)	0.601		
Poor differentiation	2.902 (1.612–5.223)	< 0.001	2.263 (1.058–4.843)	0.035
Tumour size	1.008 (1.001–1.015)	0.020		
T3/4	31.426 (4.378–225.571)	< 0.001	17.216 (2.367–125.235)	0.005
LVP	2.259 (1.385–3.682)	0.001		
Perineural invasion	2.713 (1.477–4.983)	0.001		
Mucinous	1.777 (1.005–3.143)	0.048		
LN yield ≥ 20	0.510 (0.289–0.903)	0.021	0.358 (0.170–0.756)	0.007

*HR* hazard ratio, *ASA* American Society of Anaesthesiologist grade, *CCI* Charlson co-morbidity index, *CEA* carcino-embryonic antigen, *LVP* lymphovascular permeation

HR > 1 signifies increased likelihood of disease recurrence

*The multivariate analysis only included significant parameters from the univariate analysis

### Overall survival

**Fig. 2:**
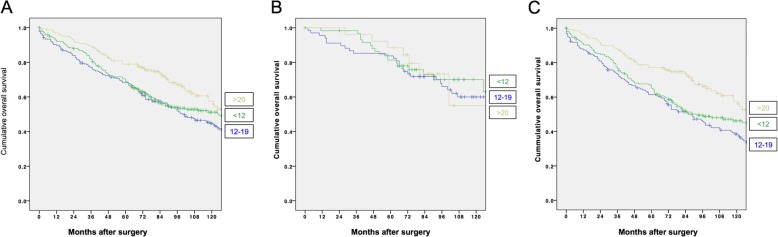
Overall survival curves for **a** overall, **b** stage I disease and **c** stage II disease

The OS according to the lymph node yield was shown in Fig. 2 a. LN≥20 lymph nodes had significantly better OS (*p* = 0.036). Figure 2 b and c showed the OS of stage I and II diseases, respectively. Similar to the DFS, the survival benefit from a higher lymph node yield was mainly seen in those with stage II diseases. The 5-year OS was 68.2%, 70.4% and 78.9% for LN<12, LN12-19 and LN≥20, respectively (Table 2). The 10-year OS was 44.8%, 51.2% and 57.5% for LN<12, LN12-19 and LN≥20, respectively.

The independent predictors of OS were high preoperative CEA level (HR 1.005; 95% CI 1.002–1.008, *p* < 0.001) lymphovascular permeation (HR 1.580; 95% CI 1.110–2.250, *p* = 0.011), ASA ≥ 3 (HR 1.907; 95% CI 1.443–2.521, *p* < 0.001), male gender (HR 1.748; 95% CI 1.328–2.299, *p* < 0.001), age (HR 1.059; 95% CI 1.042–1.076, *p* < 0.001) and T stage ≥ 3 (HR 1.428; 95% CI 1.022–1.995, *p* = 0.037) (Table 4).

**Table 4:** The univariate and multivariate analysis of various clinic-pathological factors, using overall survival as the dependent variable

	Univariate HR	*p*	*Multivariate HR	*p*
Age	1.053 (1.041–1.066)	< 0.001	1.059 (1.042–1.076)	< 0.001
Male	1.449 (1.157–1.816)	0.001	1.748 (1.328–2.299)	< 0.001
ASA ≥ 3	2.480 (1.972–3.118)	< 0.001	1.907 (1.443–2.521)	< 0.001
Year of surgery 2003–2007	1.129 (0.894–1.428)	0.308		
Preoperative CEA	1.004 (1.001–1.006)	0.005	1.005 (1.002–1.008)	< 0.001
CCI	1.364 (1.205–1.543)	< 0.001		
Obstruction	1.482 (1.133–1.938)	0.004		
Perforation	2.463 (1.581–3.837)	< 0.001		
Emergency surgery	1.953 (1.498–2.547)	< 0.001		
Laparoscopic approach	0.630 (0.502–0.790)	< 0.001		
Right-sided resection	1.364 (1.094–1.700)	0.006		
Poor differentiation	1.127 (0.716–1.774)	0.605		
Tumour size	1.020 (0.908–1.145)	0.741		
T3/4	1.738 (1.291–2.340)	< 0.001	1.428 (1.022–1.995)	0.037
LVP	1.634 (1.227–2.176)	0.001	1.580 (1.110–2.250)	0.011
Perineural invasion	1.759 (1.185–2.611)	0.005		
Mucinous	1.124 (0.794–1.925)	0.509		
LN yield ≥20	0.677 (0.513–0.894)	0.006		

*HR* hazard ratio, *ASA* American Society of Anaesthesiologist grade, *CCI* Charlson co-morbidity index, *CEA* carcino-embryonic antigen, *LVP* lymphovascular permeation

HR > 1 signifies increased likelihood of mortality

*The multivariate analysis only included significant parameters from the univariate analysis

## Discussion

Adequate lymph node yield is vital for accurate staging, determining prognosis and ascertaining the need for adjuvant treatment. The American Society of Clinical Oncology (ASCO) recommends the use of adjuvant chemotherapy in patients with stage II diseases and have a lymph node yield of less than 12 [8]. Adequate lymph node sampling was also frequently seen as an indicator of oncological clearance and cancer surgery quality [9, 10].

However, lymph node yield is affected by a multitude of factors. Apart from technical factors like the extent of resection, it also depends on factors that were not controllable by surgeons. Studies have shown that a higher lymph node yield was more likely seen in young age, right-sided resection, advanced T and N stage and greater tumour size [11–15]. The lymph node yield within an institution was also shown to be increasing at a rate of 2–3% per year [16].

Yet, lymph node yield was not only important for accurate staging. It was shown to correlate with survival in colon cancer patients, with those having a better survival when the lymph node yield was higher [4, 5, 17–21]. The logical way to explain this was a stage migration effect, i.e. more patients would be upstaged to stage III diseases if more lymph nodes were sampled [22]. However, this was disproved by previous studies. A Canadian population-based study showed that higher lymph node yield was not associated with an increase in the rate of node-positive disease [23]. Parsons and van Erning both found that the lymph node yield had increased considerably through the years but the proportion of stage III diseases remained similar [24, 25]. Storli compared hospitals with different surgical radicality and specimen evaluation methods. Despite an increase in the lymph node yield in centres with more aggressive resection and more dedicated pathologists, the percentage of stage III diseases was similar [26]. These showed that improved survival could not be explained by an upstaging effect.

Another way to explain this was the lymph node yield could be a representation of the underlying immunological response to cancer [27]. Advanced and bigger tumours are more likely to elicit a greater immunological response and hence render the lymph nodes easier to be found. An inherent better immunological response may be the answer as to why patients with more lymph node sampled had better survival. The association between microsatellite instability and lymph node yield has also been explored. Adequate lymph node harvest was more likely seen in tumours with microsatellite instability, with an odds ratio of 2.3 to 2.5 [28, 29]. While tumours with microsatellite instability were known to be associated with a good prognosis, whether this is the answer as to why patients with a greater lymph node yield had better survival remains to be elucidated [30–33].

Surgeons often strive to improve cancer control by adequate removal of the lymphatics draining the tumour-bearing colon. Yet, current evidence did not support an overzealous approach. Excessive longitudinal resection, albeit with higher lymph node yield, has no impact on survival outcomes [34]. Also, the value of removing apical lymph nodes by central vascular ligation, as in complete mesocolic excision, is subjected to debate. The evidence was still conflicting at present [6]. The Japan Society for Cancer of the Colon and Rectum recommends apical lymph node removal for advanced disease [35]. Despite that, central vascular ligation and apical lymph node removal were not shown to have an impact on survival and this represents an area that warrants further research [36].

The term inadequate lymph node yield is often misleading. A study had shown a lower lymph node yield, e.g. nine, as adequate for staging [3]. While the importance of adequate oncological clearance should not be understated, the absolute number of lymph node harvested may just be a mere reflection of underlying body immune response. The current study showed an improved survival was associated with the lymph node yield of greater than 20. The causality between lymph node yield and survival was not proven. However, a higher lymph node yield could be seen as a prognostic marker. The survival outcomes of patients with lymph node yield less than 12 were similar to those between 12 and 19. During the study period, there was a gradual adoption of the laparoscopic approach but the principle of colon cancer resection remained unchanged. The year of diagnosis was associated with a difference in lymph node yield but did not have an impact on survival outcomes. Lymph node yield below 12 per se, after adequate resection adhering to the principles of oncological clearance and diligent pathological examination, should not be seen as a risk factor. On the other hand, a lower lymph node yield associated with inadequate surgical resection poses a risk of under-staging and should follow the recommendation of ASCO and consider adjuvant treatment.

This study was limited by its retrospective nature and therefore bias could exist between the different groups. There were differences in the clinic-pathological characteristics between the three groups. Patients in the LN≥20 group were younger, had a lower ASA grade, had bigger tumours and were less likely to be operated with the open approach. Prior studies showed that bigger tumours and younger age were associated with a higher lymph node yield [11, 16]. Surgeons in the unit followed a standardized operative approach. Nevertheless, there remained a possibility that young and fit patients with bigger tumours were treated more aggressively. Likewise, there was a possibility that patients in the LN≥20 group were operated by experienced surgeons who were proficient in the laparoscopic approach. Nevertheless, none of these was predictors of DFS. ASA grade and age certainly affected the analysis of OS. By performing multivariate analysis of the DFS, the effect was minimized.

There was also a possibility that a difference in survival was not detected between the groups of lymph node yield less than 12 and between 12 and 19 due to the relative small sample size. The quality of the mesocolon was not graded by surgeons or pathologists and was not analysed in this study. A better mesocolon quality may be associated with higher lymph node yield and hence served as a potential confounding factor for survival outcomes.

## Conclusion

In conclusion, patients with lymph node yield greater than 20 were associated with better survival. This could be seen as a prognostic factor for better oncological outcomes. Those with lymph node yield less than 12 had similar survival outcomes as those with more than 12 but less than 20. Given adequate surgery and pathological examination, lymph node yield less than 12 should not be seen as a poor prognostic factor.

## Data Availability

The datasets generated and/or analysed during the current study are available from the corresponding author on request.
